# Optical Pump–Terahertz Probe Diagnostics of the Carrier Dynamics in Diamonds

**DOI:** 10.3390/ma17010119

**Published:** 2023-12-26

**Authors:** Vladislava Bulgakova, Pavel Chizhov, Alexander Ushakov, Pavel Ratnikov, Yuri Goncharov, Artem Martyanov, Vitali Kononenko, Sergey Savin, Ilya Golovnin, Vitaly Konov, Sergey Garnov

**Affiliations:** 1 Prokhorov General Physics Institute of the Russian Academy of Sciences, 119991 Moscow, Russia; pvch@kapella.gpi.ru (P.C.);; 2 Moscow Institute of Physics and Technology, 141701 Dolgoprudny, Russia; 3 Nanocenter MIREA, MIREA—Russian Technological University, 119454 Moscow, Russia; 4 Faculty of Physics, Lomonosov Moscow State University, 119991 Moscow, Russia

**Keywords:** optical pump–terahertz probe (OPTP), diamond, silicon, composites, nitrogen, boron, photoinduced charge carriers

## Abstract

Diamond is a promising material for terahertz applications. In this work, we use a non-invasive optical pump–terahertz probe method to experimentally study the photoinduced carrier dynamics in doped diamond monocrystals and a new diamond-silicon composite. The chemical vapor deposited diamond substrate with embedded silicon microparticles showed two photoinduced carrier lifetimes (short lifetime on the order of 4 ps and long lifetime on the order of 200 ps). The short lifetime is several times less than in boron-doped diamonds and nitrogen-doped diamonds which were grown using a high temperature–high pressure technique. The observed phenomenon is explained by the transport of photoexcited carriers across the silicon–diamond interface, resulting in dual relaxation dynamics. The observed phenomenon could be used for ultrafast flexible terahertz modulation.

## 1. Introduction

An increasing number of important fundamental and applied tasks, such as materials studies, industrial inspection, medical diagnosis, and the next generation of telecommunication devices, aim to use terahertz (THz) electromagnetic waves [1,2,3,4,5,6,7]. But still, THz frequencies remain among the least exploited in the electromagnetic spectrum, largely due to the lack of powerful and compact sources. One of the most promising candidates for such a source is photoconductive antennas (PCAs) based on high-field transport of charge carriers generated in the antenna substrate by femtosecond optical pulses [8,9]. PCAs with a radiating area larger than the wavelength of THz radiation are called large-aperture photoconductive antennas (LAPCAs) [8,10]. LAPCAs have a predominance of low-frequency components in the pulse spectrum due to the quasi-half-periodicity of the pulse. Therefore, these sources are of interest for many applications [11,12], including ionization of Rydberg atoms and high-frequency lasing [13,14]. Improving the efficiency of THz LAPCAs through better designs or the use of novel materials is one of the main goals of ongoing research on such generators. The main role in the generation of THz radiation by LAPCAs is played by the properties of the semiconductor substrates, such as the electric field breakdown threshold, the lifetime of the non-equilibrium carriers, and their mobility. Due to the high electric field breakdown threshold, one of the prospective substrates for a LAPCA is diamond.

Diamond has a band gap of 5.46 eV, which requires deep-ultraviolet radiation for efficient single-photon excitation. However, it has been demonstrated to be a LAPCA source with a laser excitation wavelength of 248 nm [15]. To overcome the band gap limitation, it was shown that the diamond electronic system could be excited by absorption on nitrogen defects [16] and boron defects [17]. The introduction of defects into the crystal lattice is also a way to shorten photocarrier lifetimes [18], which is important for detector photoconductive antennas. All-optical terahertz modulators are also devices where the photocarrier recombination dynamics is of great importance [19,20,21]. In addition, precisely engineered synthetic diamond materials are now widely available through the use of various chemical vapor deposition (CVD) techniques [22]. In particular, micrometer-thin diamond membranes can be produced for various applications such as masks for X-ray lithography, MEMS, pressure sensors, etc. [23,24,25,26]. In addition, in the process of CVD synthesis, various micro- and nanoparticles can be embedded in the bulk of diamond layers [27], which can lead, for example, to the formation of highly X-ray luminescent diamond membranes [28]. Thus, the CVD technique is quite flexible for the formation of novel diamond-based materials for various applications, including the fabrication of THz radiation sources.

The determination of the lifetime of non-equilibrium carriers and their mobility in the substrate of LAPCAs can be performed by a non-contact and non-destructive method of optical pump–terahertz probe (OPTP) [29]. In OPTP experiments, an ultrashort laser pulse generates free charge carriers while broadband THz pulses (typically in the range of 0.3–3 THz, corresponding to photon energies of 1.2 meV to 12.4 meV) are used to probe the transmission of the sample. Unlike traditional optical pump–optical probe experiments, the OPTP technique allows the measurement of the time-dependent electric field of the probe pulse [29]. Through Fourier analysis, the complete complex absorption spectrum can be obtained, providing details of amplitude and phase shift changes. When a semiconductor is exposed to THz radiation, the alternating electric field E(ω) of the radiation can induce the motion of carriers and excitations (such as free electrons, holes, surface plasmons, excitons, and polarons) and interact with optical phonons [30]. The THz frequency range is comparable to the scattering rates of charge carriers in inorganic semiconductors [31,32]. Therefore, THz spectroscopy allows the study of low-energy electronic processes in semiconductors and provides valuable insight into the electrical conductivity of semiconductors [33,34].

The electron–hole plasma behavior in diamond is not fully understood. Only a few papers are known to have reported carrier lifetime data for diamonds of unspecified quality and purity [35,36]. According to these works, the typical recombination time of the photoexcited plasma is about 10–100 ps. In this paper, we compare the recombination dynamics of photocarriers excited on different impurities in diamond substrates using the OPTP technique. In addition to monocrystalline diamond, we investigated a novel class of diamond composites with embedded Si microparticles and found interesting relaxation dynamics consisting of fast relaxation on the time scale of a few picoseconds and a slow tail lasting hundreds of picoseconds.

## 2. Materials and Methods

**Figure 1:**
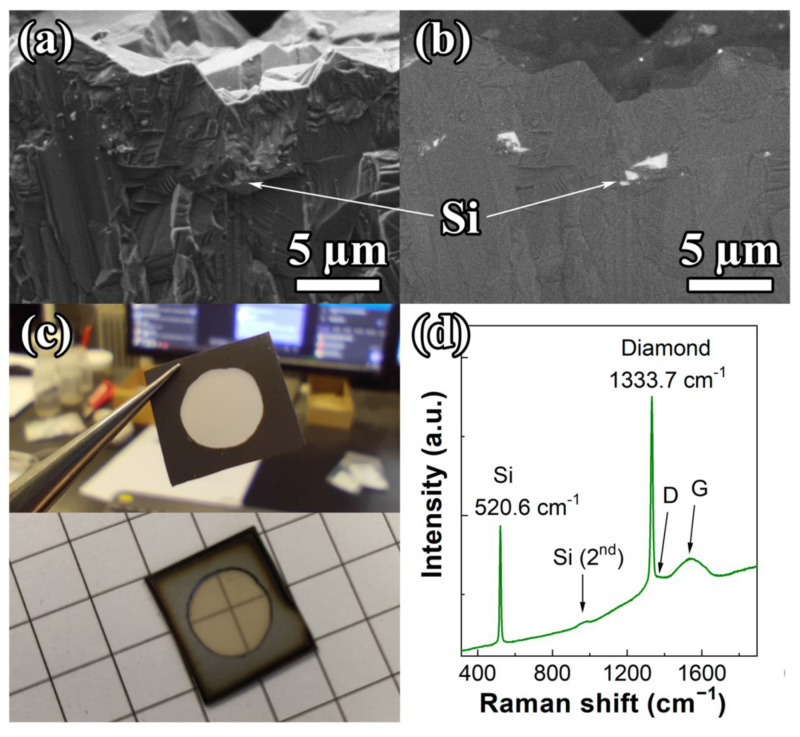
Diamond membrane on silicon substrate: cross-sectional SEM images taken in SE (**a**) and BSE (**b**) modes, photos (**c**), typical Raman spectrum of the diamond membrane over a Si particle (**d**).

A CVD diamond sample was formed in two steps using microwave plasma chemical vapor deposition (MPCVD, ARDIS-100, 2.45 GHz, Optosystems LLC (Moscow, Russia)) [37] in a hydrogen–methane gas mixtures. In the first step, silicon <100> substrate 10 × 10 mm was seeded with nanodiamond particles (particle sizes 3–7 nm, zeta potential >+50 mV, Cardiff University [38]), which became nucleation centers for the growth of the first 15 µm thick polycrystalline MPCVD diamond layer. In the second step, the surface of this grown polycrystalline diamond film was spin-coated (3000 rpm, 0.5 min) with a milled silicon suspension in dimethylsulfoxide (DMSO, with a concentration of 10 mg/mL). A second diamond layer with a 4 µm thickness was grown using MPCVD to encapsulate the Si particles in the diamond substrate completely. The final thickness of the PCD film was 19 µm. The sample was grown using the methane and hydrogen gas mixture with a fixed total gas flow at 500 sccm (methane content of 4%), pressure of 75 Torr, microwave power of 4.5 kW, and temperature of 840 ${}^{{}^{\circ}}$C, as measured by a two-color pyrometer METIS M322 (SensorTherm GmbH, Steinbach, Germany) with accuracy ±10 ${}^{{}^{\circ}}$C. The silicon substrate was partially removed by chemical etching in a mixture of concentrated acids HF:HNO_3_ (3:1, 40 ${}^{{}^{\circ}}$C, 1–2 min) to form a diamond membrane (Figure 1a). The morphology of the synthesized sample was examined using a Tescan MIRA3 scanning electron microscope (SEM, Tescan, Brno, Czech Republic). The secondary-emission (SE) imaging mode of the SEM shows the high-quality polycrystalline structure of the diamond membrane, with a grain size of 5–10 µm (Figure 1a). In backscattered electron (BSE) mode, which enhances the contrast between different phases due to the high Z-contrast between carbon and silicon, the encapsulated Si particles are clearly visible as light contrast areas (Figure 1b). Silicon particles of 100 nm–2 µm size lie at distances of a few microns from each other. The phase composition of the film was analyzed at room temperature with micro-Raman spectroscopy using a Horiba Jobin Yvon LabRAM HR800 (HORIBA FRANCE SAS, Vénissieux, France) equipped with a diode-pumped solid-state laser ($\lambda_{exc}$ = 473 nm). The spectrometer operates in a confocal mode, while the laser beam is focused in a spot of 2 µm in diameter on the membrane surface. The Raman spectrum of the diamond membrane had the following features which are common for polycrystalline diamond films: a sharp diamond peak at 1333.7 cm${}^{-1}$ (FWHM = 4.3 cm${}^{-1}$), and two wide bands from D- and G-peaks at 1350 and 1580 cm${}^{-1}$, respectively, from graphitic carbon (Figure 1d). The shift (by 1.2 cm${}^{-1}$) and slight broadening of the diamond line are typical for polycrystalline CVD diamond films and are associated with defects in the crystal structure. The peak near 520.6 cm${}^{-1}$ corresponds to silicon. Taking into account the instrumental error (∼0.5 cm${}^{-1}$), we cannot confirm the shift in silicon peak position, caused by tensile stress due to the difference in the thermal expansion coefficients of diamond and silicon.

**Figure 2:**
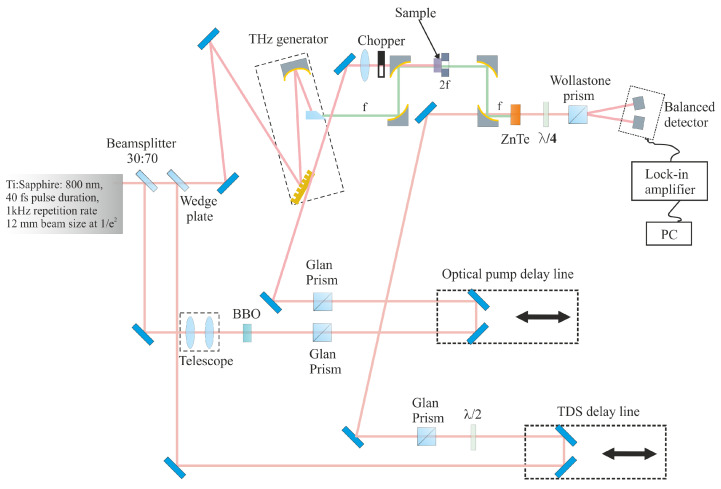
Scheme of the experimental setup of OPTP experiments.

In experiments on optical pumping–terahertz probing (Figure 2), the output beam of a titanium–sapphire laser system (central wavelength 800 nm, pulse energy 3 mJ, pulse duration 40–150 fs, radiation spot diameter 12 mm at the level 1/e${}^{2}$, pulse repetition rate 1 kHz) is divided into three beams using a beam splitter (splitting the radiation in the ratio 70/30 (transmission/reflection)) and a wedge-shaped beam splitter (splitting the radiation in the ratio 96/4 (transmission/reflection)). The main part of the radiation (67%), after passing through the beam splitter and the wedge plate, is used to generate terahertz radiation. The second beam (30%), reflected by the beam splitter, is used to excite charge carriers in the photoconductive materials under study. The third beam (3%), reflected from the wedge plate, is used to detect terahertz radiation. The main part of the laser radiation is directed to the terahertz pulse generator. The operating principle of the generator is based on the optical rectification of femtosecond laser pulses with an inclined intensity front in a nonlinear LiNbO${}_{3}$ crystal [39]. The electric field strength of the emitted terahertz pulses is up to 30 kV/cm. The output THz beam (used as a probing beam) is collimated and focused by parabolic mirrors onto the central part of the investigated semiconductor samples with a spot size of 3 mm. The radiation transmitted through the test sample is collimated by a parabolic mirror and focused into a zinc telluride crystal (ZnTe) used for THz pulse detection. Terahertz radiation is detected by electro-optical gating in the zinc telluride crystal. The effect of induced birefringence occurs in the crystal at the moment of arrival of a terahertz radiation pulse. The weakest part of the laser radiation reflected by the wedge plate is used for its registration. This beam is directed to a mechanized translation stage with a corner reflector and then passes through a hole in the last focusing parabolic mirror onto a zinc telluride crystal. The state of polarization of the detecting beam changes when it passes through a zinc telluride crystal with induced birefringence, and the resulting phase delay between the ordinary and extraordinary waves depends linearly on the magnitude of the terahertz electric field acting on the crystal at the moment of passage of the detecting laser pulse. To register the electric field strength of a terahertz pulse from the degree of depolarization of the detecting radiation, a scheme is implemented with a balanced detector consisting of a quarter-wave phase plate oriented with a fast axis at $45^{{}^{\circ}}$ to the direction of polarization of the laser radiation, a Wollaston prism, separating two orthogonally polarized components of the detecting radiation, and two photodiodes. The difference in the signals in the photodiodes is proportional to the magnitude of the THz electric field. The signal from the balanced detector is recorded on the lock-in amplifier, with registration at the repetition rate of laser pulses. A half-wave plate and a Glan prism are used to adjust the power of the detecting beam. Diaphragms ensure the passage of the detecting beam and protection against parasitic illumination of the balanced detector. The part of the laser radiation reflected by the beam splitter is used for the optical excitation of charge carriers. The radiation is directed to another motorized translation stage with a corner reflector, and then to the surface of the test sample. By moving the corner reflector, the length of the pump radiation path and, thus, the time delay between the moments of arrival of the pump radiation pulse and the terahertz probing pulse at the sample surface is controlled. A beta-barium borate (BBO) crystal (10 × 10 × 0.2 mm${}^{3}$, I-type) is embedded in the pump beam to convert a part of the radiation into the second harmonic to excite photoconductive carriers in nitrogen-doped diamond samples. To boost the conversion efficiency the beam is reduced twofold in diameter by a telescope. The pump radiation is expanded by a lens to a diameter of 13 mm at the 1/e${}^{2}$ level (at the waist of the terahertz radiation). The energy density of the optical radiation of the pump can be varied by a system of two linear polarizers (Glan prisms), by changing the angle of rotation of the Glan prism, installed on the optical path in front of the second Glan prism, fixed in one position. Using written software, the mechanized stages are controlled and a signal is recorded from a lock-in amplifier. This makes it possible to automatically measure the time dependencies of the electric field strength of a terahertz pulse transmitted through a sample with a fixed delay in the arrival of the terahertz pulse relative to the pulse of optical excitation of photocarriers. Therefore, this setup makes it possible to estimate the lifetimes of photoexcited charge carriers and measure the transmission spectra of terahertz radiation of the photoexcited sample.

For characterization of the diamond sample’s absorption, an Avaspec-2048 fiber-optic spectrometer (Avantes, Apeldoorn, The Netherlands) for measurements of the transmission spectra of the samples was used. The spectral range of the spectrometer is from 230 to 1100 nm, and the spectral resolution is 2.4 nm.

## 3. Results

**Figure 3:**
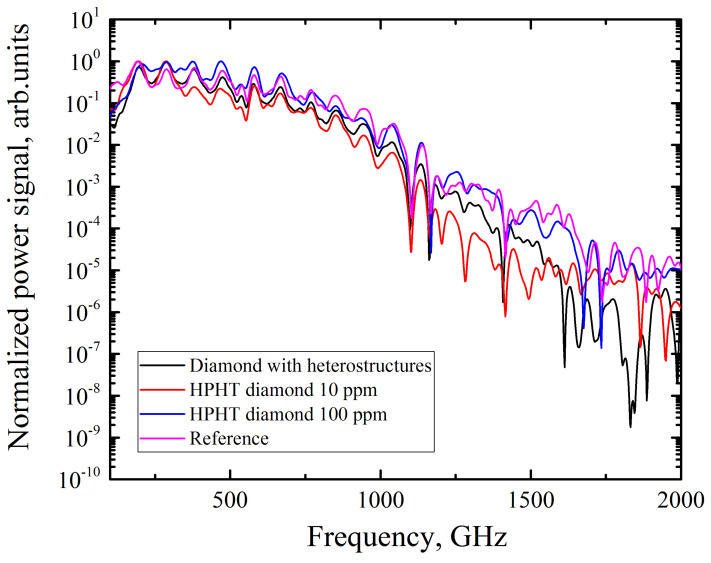
Power spectra of terahertz pulses passed through the diamond with embedded Si particles, HPHT diamond with a nitrogen impurities concentration of 10 ppm, and HPHT diamond with a nitrogen impurities concentration of 100 ppm.

To compare the dynamics of photocarrier relaxation, boron-doped diamond samples were excited by fundamental-harmonic laser radiation, while nitrogen-doped diamonds were exposed to second-harmonic laser radiation (which connected with absorption of samples, that will be show below); the sample with Si particles was pumped with both wavelengths. The initial spectrum of the THz pulses source and the spectra of the THz pulses transmitted through two types of high temperature–high pressure (HPHT) grown diamonds and the Si–diamond composite are shown in Figure 3. The character of the changes due to transmission through the diamond samples allows us to assume that there are no resonance lines in the studied samples in the considered spectral region of 0–1.5 THz.

**Figure 4:**
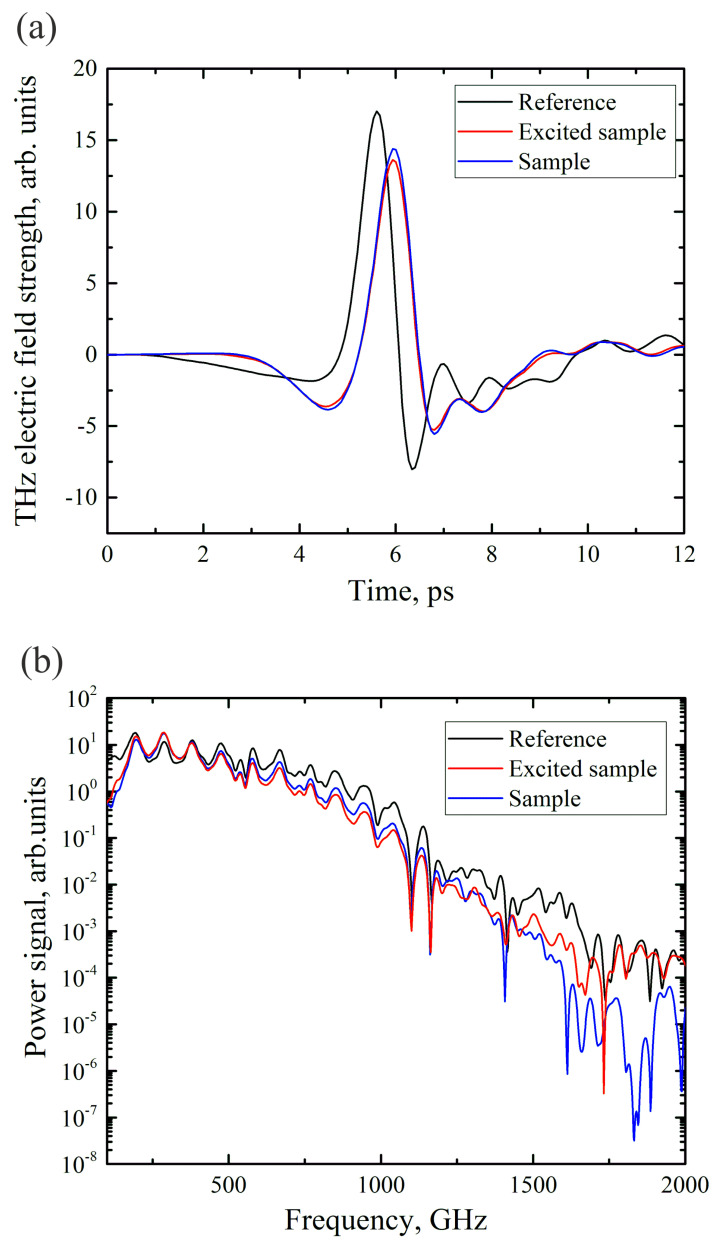
(**a**) Waveforms of THz pulses in a free space (reference), passed through Si–diamond composite (sample), and passed through Si–diamond composite under optical excitation (excited sample); (**b**) power spectra of terahertz pulses.

The measured THz pulse electric field strength waveforms and corresponding spectra for the reference THz pulse, for the diamond with embedded Si particles under photoexcitation (167 µJ/cm${}^{2}$ at 800 nm) and for the same sample without excitation are shown in Figure 4. It can be seen that there is a 15 percent decrease in the amplitude of the transmitted THz signal for the unexcited sample and a 20 percent decrease for the excited sample. However, no significant difference is observed in the spectra of the THz pulses transmitted through the Si–diamond composite in the case of photoexcitation. Therefore, data on the dynamics of photoinduced carriers can be obtained by following the maximum electric field strength of the THz signal, since there is no change in the temporal shape of the electric field strength during photoexcitation.

The OPTP measures the dynamical properties by introducing the optical delay between the optical pump and THz probe beam. This technique allows us to track the dynamics of photoinduced charges as a function of the pump–probe delay time. The THz electric field strength transmitted through the sample without photoexcitation $E_{off}$ and under photoexcitation $E_{on}$ was measured to calculate the relative change in the THz transmission (modulation depth) $\Delta E/E\;=\;\Delta T/T\;=\;\left(E_{on}-E_{off}\right)/E_{off}$. Irradiation of a semiconductor with light leads to the appearance of an excess concentration of charge carriers (injection). The process of recombination of photoexcited charge carriers (when the lighting is turned off) is described by an exponential dependence on time (in the case of a low injection level). An exponential decay of the excited state is the most common dependence which can be observed in pump–probe diagnostics, photoluminescence studies, etc. The fit can consist of the sum of several exponents correlating to different relaxation processes each with its own dynamics and temporal constant. In our case, the boron- and nitrogen-doped samples demonstrated only one dominant relaxation time, while the diamond sample with embedded Si possessed two different temporal constants.

**Figure 5:**
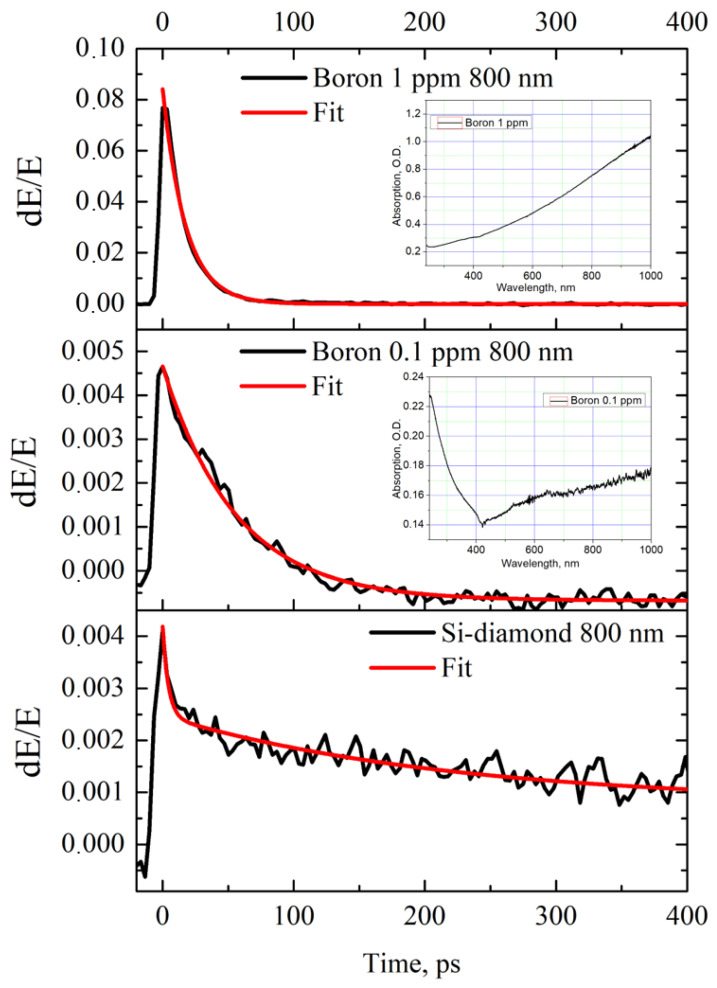
The relative transmission as a function of pump–probe delay time, for HPHT boron-doped (1 ppm and 0.1 ppm) diamonds and diamond with silicon particles (at 800 nm excitation wavelength). Optical absorption spectra of diamond samples are given in insets.

**Figure 6:**
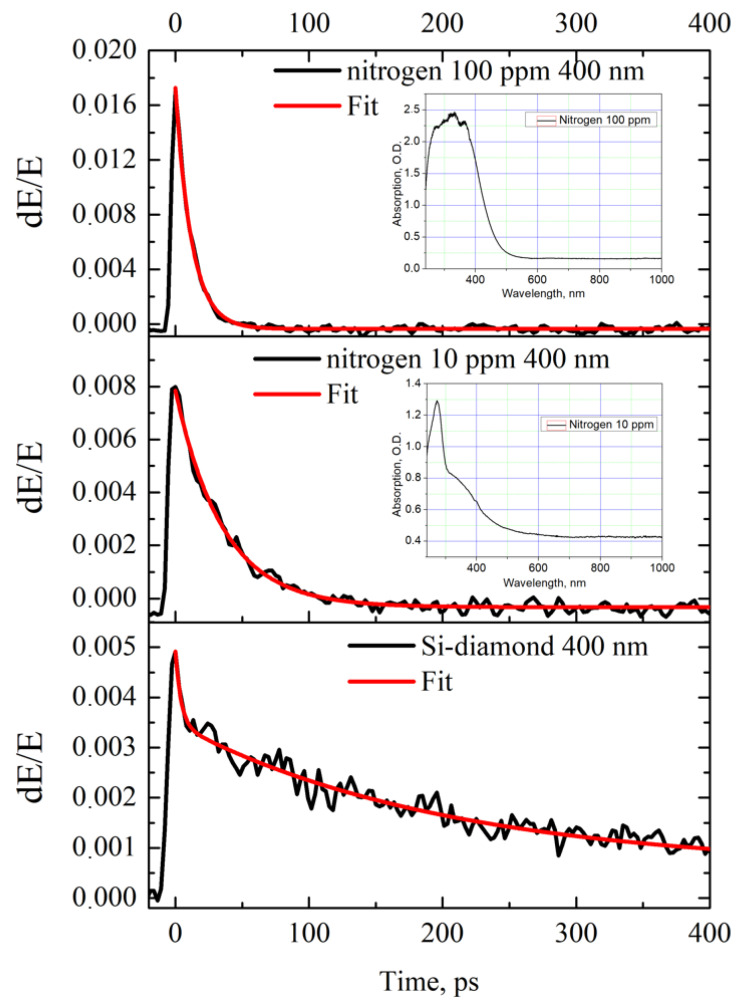
The relative transmission as a function of pump–probe delay time, for HPHT nitrogen-doped (100 ppm and 10 ppm) diamonds (at 400 nm excitation wavelength). Optical absorption spectra of diamond samples are given in insets.

In Figure 5 and Figure 6, the temporal dynamics of the change in the transmission of the photoexcited diamond samples are given. The samples with nitrogen impurities demonstrate rather full recombination of the photoexcited carriers on the tens of picosecond time scale (Figure 6). It is worth noting the faster relaxation in the case of a sample with more defects with 100 ppm nitrogen concentration (t_100ppm_ = 10 ± 1 ps) as compared to that of the one with 10 ppm (t_10ppm_ = 25 ± 1 ps). The more doped sample also demonstrates a deeper modulation contrast when excited with the same pump beam fluence (1.5 percent vs. 0.8 percent for 10 µJ/cm${}^{2}$ at 400 nm). The samples with boron impurities (1 ppm and 0.1 ppm) show similar relaxation behavior (Figure 5). The more doped sample possesses a faster relaxation time (1 ppm, t_1ppm_ = 17 ± 1 ps) compared to the less doped one (1 ppm, t_0.1ppm_ = 55 ± 2 ps). The modulation depth is 7.5 percent vs. 0.45 percent for 10 µJ/cm${}^{2}$ at 800 nm, correspondingly.

The most interesting dynamics in the relaxation is observed for the sample with embedded Si particles. It has prominent “hot”-carrier relaxation with t${}_{Sifast}$ = 4 ± 0.5 ps that corresponds to more than a third of the full transmission change. The subsequent slow relaxation dynamics is described by the time constant t${}_{Sislow}$ = 220 ± 100 ps. An interpretation of the observed phenomenon will be presented below.

**Figure 7:**
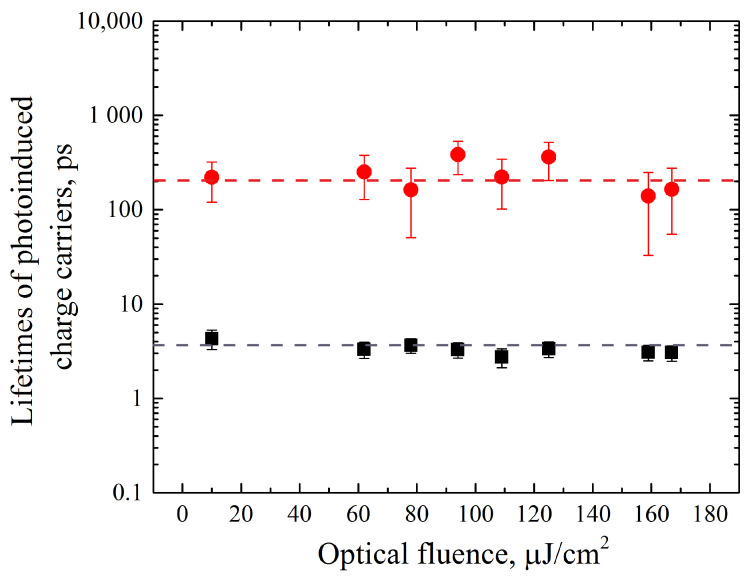
Lifetime of photoinduced charge carriers as a function of the optical fluence for 800 nm excitation wavelength: the first relaxation time—black dots; the second relaxation time—red dots.

**Figure 8:**
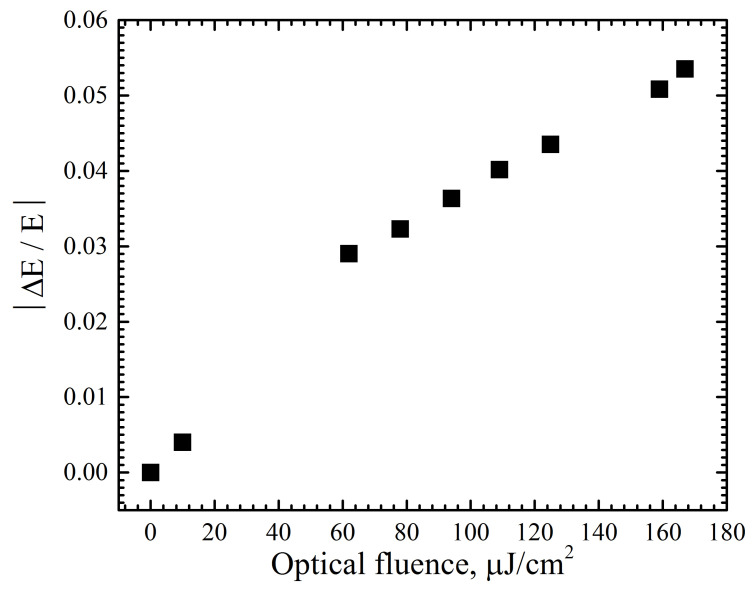
The dependence of the maximum value of the relative transmission module of the terahertz signal on the optical fluence.

As can be seen from Figure 7, the fast and slow temporal constants do not change when the optical fluence changes. Since the mobility of charge carriers is directly proportional to the free-path time (which is equivalent to the carrier lifetime), it can be concluded that the mobility does not change within the experimental values of the optical fluence. From Figure 8 it can be concluded that the modulus of transmittance changes practically linearly with increasing optical fluence in the 60 to 170 µJ/cm${}^{2}$ region. However, this is not the case for lower pump energies. It can be noted that the relative change in transmission is lower for this Si–diamond composite (0.4 percent and 0.5 percent for 10 µJ/cm${}^{2}$ at 800 nm and 400 nm, respectively). This fact could be connected with the minor volume of the structured area as compared to the bulk size of the boron- and nitrogen-doped diamond samples where the absorption of the laser pump beam occurs.

## 4. Discussion

Since the photon energy of the laser radiation ℏω=1.55 eV exceeds the indirect band gap of silicon $E_{gSi}=1.12$ eV, but is smaller than that of diamond $E_{gd}=5.46$ eV, the light dissipates in silicon by single-photon absorption, while diamond remains completely transparent. The relaxation time of bulk silicon is known to be several nanoseconds. During this time, electrons and holes born in silicon have time to reach the micron-scale silicon–diamond interface. Therefore, all the dynamics are determined by the processes at the silicon–diamond interface.

The two-step relaxation observed in the experiment can be explained as follows. First, the holes generated in the silicon particle move to its boundary: the ratio of the edges of the valence bands of silicon and diamond is such that it is energetically favorable for them to leave the silicon and enter the diamond. The generated electrons are retained inside the silicon by high potential barriers. As a result, some holes are localized at the shallow defects in the diamond, which is rather imperfect in the vicinity of the silicon particle. These holes attract the electrons and recombine with them. We estimate that this process takes a few picoseconds, which explains the observed relaxation time of ∼4 ps.

The rest of the holes released in the diamond are attracted back to the silicon–diamond interfaces by the uncompensated charge of the electrons in the silicon, which are also attracted to them. As a result, a doubly charged layer is formed along the interface. The probability of recombination during the spatial separation of electrons and holes decreases due to a decrease in the overlap integral of the electron and hole wave functions. We estimate that the probability of recombination in this case decreases by an order of magnitude compared to recombination in bulk diamond, for which the relaxation time is 16–20 ps. We believe this mechanism explains the relaxation time of hundreds of picoseconds.

## 5. Conclusions

In conclusion, we have measured the photocarrier relaxation dynamics in diamond samples with nitrogen, boron, and Si particle impurities. The general behavior of shortening the photocarrier relaxation time for a more defective material is observed. The modulation depth in the THz transmission strongly depends on the impurity level and the volume where the absorption takes place. The introduction of silicon microparticles into the diamond sample leads to dual relaxation dynamics. The mechanism of this dual recombination of photoexcited carriers in the diamond–silicon composite has been proposed. It implies that carriers are generated by one-photon absorption of silicon and then transferred from silicon to the diamond crystal lattice. As a result of carrier transport across the silicon–diamond interface, recombination actually occurs in diamond and is delayed by the charge separation effect. The observed phenomenon could be used for ultrafast flexible THz wave modulation.

## 6. Patents

The patent number RU221535U1 declares the experimental setup described in this work.

## Data Availability

Data available on request.

## Abbreviations

The following abbreviations are used in this manuscript:

THz	Terahertz
PCA	Photoconductive antenna
LAPCA	Large-aperture photoconductive antenna
OPTP	Optical pump–Terahertz probe
CVD	Chemical vapor deposition
MEMS	Micro-electro-mechanical system
MPCVD	Microwave plasma chemical vapor deposition
GHz	Gigahertz
DMSO	Dimethylsulfoxide
Si	Silicon
PCD	Polycrystalline diamond
SEM	Scanning electron microscope
SE	Secondary emission
BSE	Backscattered electron
LiNbO${}_{3}$	Lithium niobate
ZnTe	Zinc telluride
BBO	Beta-barium borate
HPHT	High pressure–high temperature
